# Urosepsis due to obstructive stones: Epidemiological data from a population‐based study in Sweden

**DOI:** 10.1002/bco2.70158

**Published:** 2026-02-16

**Authors:** Hjalmar Svensson, Lars Grenabo, Klas Lindqvist, Erik Sörstedt, Jonas Hugosson

**Affiliations:** ^1^ Department of Urology, Sahlgrenska Academy University of Gothenburg Gothenburg Sweden; ^2^ Urological Clinic, Carlanderska Hospital Gothenburg Sweden; ^3^ Department of Urology Sahlgrenska University Hospital Gothenburg Sweden; ^4^ Department of Infectious Diseases Sahlgrenska University Hospital Gothenburg Sweden; ^5^ Department of Infectious Diseases, Sahlgrenska Academy University of Gothenburg Gothenburg Sweden

**Keywords:** epidemiology, sepsis, urinary calculi, urinary tract infection, urolithiasis

## Abstract

**Objective:**

The study's aim is to provide a population‐based description of the incidence, epidemiology, and clinical course of urinary tract infection or sepsis caused by ureteric stone obstruction. Although being a life‐threatening condition, there have been few epidemiological reports on this disease.

**Patients and Methods:**

The Swedish National Patient Register and local hospital databases were used to identify all adults discharged from inpatient care, with a combination of the International Classification of Disease 10th revision codes for urolithiasis and urinary tract infection in the Region Västra Götaland in Sweden for 2 years. Exclusion criteria were ongoing treatment for a urinary stone, nonsignificant infection and no obstruction from the stone. Medical records were reviewed to collect descriptive statistics on patient characteristics and clinical outcomes until stone‐free.

**Results:**

The register and local hospital search identified 702 patients with a predefined combination of diagnostic codes; 387 were excluded, leaving 315 for analysis. The incidence of acute obstructive urinary tract infection was 11.8 per 100 000 inhabitants per year. The median age was 68 years, and 176 (56%) were women. Fifty patients (16%) required intensive care unit admission and eight (3%) died. Four of these deaths were from acute septic complications, while the others died waiting for definitive stone treatment.

**Conclusion:**

Acute obstructive urinary tract infection mainly affects elderly patients and has a variable clinical course, which in severe cases demands intensive care and may even be fatal.

## 1 INTRODUCTION

Patients presenting with a combination of obstructing upper urinary tract stones and urinary tract infection (UTI) are a relatively common group in emergency departments (EDs) worldwide. This urological emergency can rapidly progress to severe septic shock and even death. However, this condition may also present as a milder disease resolved with antibiotic therapy alone.^1^, ^2^

The formation of stones in the upper urinary tract is a multifactorial process with dietary changes, weight gain and diabetes as contributing factors to its increased prevalence. For example, between 1994 and 2012, the prevalence of upper tract urolithiasis increased from 4.1% to 7.1% in women and 6.3% to 10.6% in men in the United States.^3^ Along with the increasing prevalence of urolithiasis in developed countries, urosepsis as a complication is also expected to become more common and a major challenge for healthcare systems.^4^, ^5^, ^6^

For urologists, treatment decisions in patients with obstructing stones and UTI are often based on local routines and clinical experience, as the scientific evidence is frequently derived from small studies. Empirical antibiotic therapy is routinely administered as bacterial cultures are lacking in the emergency setting, and supportive care is provided when indicated.^7^ The consensus is that the elevated pressure in the affected kidney needs to be decompressed by either a ureteric stent or percutaneous nephrostomy.^8^, ^9^

In Sweden, patients suspected to have an obstructing upper tract stone with associated infection are referred to a hospital with an ED. Empirical antibiotics are administered after laboratory testing. The type of decompression is decided by the clinician on call (typically a consultant urologist or general surgeon at the smaller hospitals) based on patient condition and the available methods on site (usually a nephrostomy tube or a double‐J stent).

As patients recover, they are discharged, and follow‐up treatment is typically coordinated by a senior urologist. Upon discharge, relevant ICD‐10 codes are registered in the medical records.

Most published studies on this subject are retrospective and focus primarily on patients who undergo surgical interventions in urologic departments after admission. To our knowledge, the only published study describing the annual incidence was published in 2013.^10^

This study aimed to explore the demographics, incidence, and outcomes of these patients in a well‐defined population.

## 2 MATERIALS AND METHODS

### 2.1 Target population, patient selection and study period

The Västra Götaland region in Sweden has a population of 1.3 million inhabitants. To identify all patients hospitalized for a combination of upper urinary tract stones and UTI in this region, we used the Swedish National Patient Register maintained by the National Board of Health and Welfare. It covers all hospitals in the country and, thus, all five emergency hospitals in the region: Sahlgrenska University Hospital Gothenburg, NÄL Regional Hospital Uddevalla, Skaraborg Regional Hospital Skövde, SÄS Regional Hospital Borås, Alingsås County Hospital and Kungälv County Hospital. The study period included all hospital admissions from 1 January 2017 to 31 December 2018.

The search criteria included adults (aged >18 years) who were discharged after hospitalization with a combination of International Classification of Disease 10th revision (ICD‐10) codes for both upper urinary tract stones and UTIs. The relevant codes included N20.0, N20.1, N20.2 and N20.9 in combination with N39.0, N10.9, N13.6 and A41.9.

To verify the register, we also searched the local databases at each of the hospitals to minimize the risk of missing cases. This procedure was carried out by local medical secretaries at the included five hospitals, with the same combination of ICD‐10 codes.

Given the likelihood that the above criteria would capture patients outside the intended study population, we applied the following exclusion criteria.

- Ongoing/recent treatment for an upper urinary tract stone: Patients admitted with a postoperative infection after elective stone surgery will receive the same combination of ICD‐10 codes but were excluded as they did not have a primary infection.
- Nonobstructing stone: Patients with a UTI or unilateral pyelonephritis in the presence of a nonobstructing peripheral kidney stone were excluded. Only cases with confirmed obstruction of the upper urinary tract caused by stones were included. Obstructive stones were defined as any of the following: stones in the ureter or stones in the renal pelvis with indirect signs of obstruction (perirenal stranding, hydronephrosis, thickening of the renal pelvis).
- No evidence of infection: In clinical practice, we notice that sometimes patients with uncomplicated acute renal colic may present with slightly elevated creatinine or C‐reactive protein (CRP) levels or positive leukocytes on urine dipsticks. These cases are sometimes incorrectly diagnosed as UTIs by nonurologists.^11^ To minimize misclassification, we excluded patients without documented fever exceeding 38° C combined with CRP‐levels less than 100 mg/L.

### 2.2 Retrospective data collection

The electronic medical records of all identified patients were retrospectively reviewed by a single consultant urologist (HS), and all patients who met the inclusion and exclusion criteria were included in the study. The reviewer had access to the electronic patient records, laboratory results (microbiological reports excluded) and radiology reports during the study period. A predefined study protocol regulated which clinical data were retrieved and entered into the database. Data protection is regulated by Swedish law, and the database was hosted at the University of Gothenburg.

Data on patient comorbidities, previous stone history, and the date of the first symptoms were extracted from the text in the patient records. Stone size (maximum diameter) was taken from the radiology report. To assess comorbidities, the patients were classified retrospectively using the Charlson comorbidity index (CCI).^12^

Data collection began at the time of the initial contact with the hospital for symptoms related to the obstructing stone. The follow‐up period continued until either the stone was treated and the patient was assessed as stone‐free, or a decision was made not to pursue treatment due to significant patient comorbidities.

### 2.3 Statistical analysis

Descriptive statistics were used to summarize the patient characteristics and parameters upon admission to the hospital. No imputation of missing data was performed. All calculations were conducted using SPSS statistical software package version 29.0 (IBM, Chicago, IL, USA). For an annual incidence number, the population estimate in the middle of the 2‐year study period (as of 31 December 2017) was used. No comparative statistical analyses on subgroups were employed as this study is retrospective and descriptive in its design with too few events to enable such analyses. With the same arguments, we did not calculate confidence intervals but used interquartile range to describe variations.

### 2.4 Ethical considerations

Ethical approval was obtained before data collection from the Swedish Ethical Review Authority (diary numbers: 2019–06320, 2023–03497‐02, and 2024–04443‐02). This authority regulates all ethical permits for scientific research in Sweden and adheres to the principles of the Declaration of Helsinki. As a purely retrospective analysis, no patient consent was required by the authority.

## 3 RESULTS

The registry search identified 476 patients, with an additional 226 cases found through local database searches during the study period using the prespecified combination of ICD‐10 codes, making a total of 702 patients for review. The missing cases in the registry search were found to be inadequately reported or recorded data to the register from two hospitals in the region. The reason for this has not been investigated but reported to the registry administration. The cases found through local database searches did not differ in number or severity from expected related to hospital size.

**FIGURE 1:**
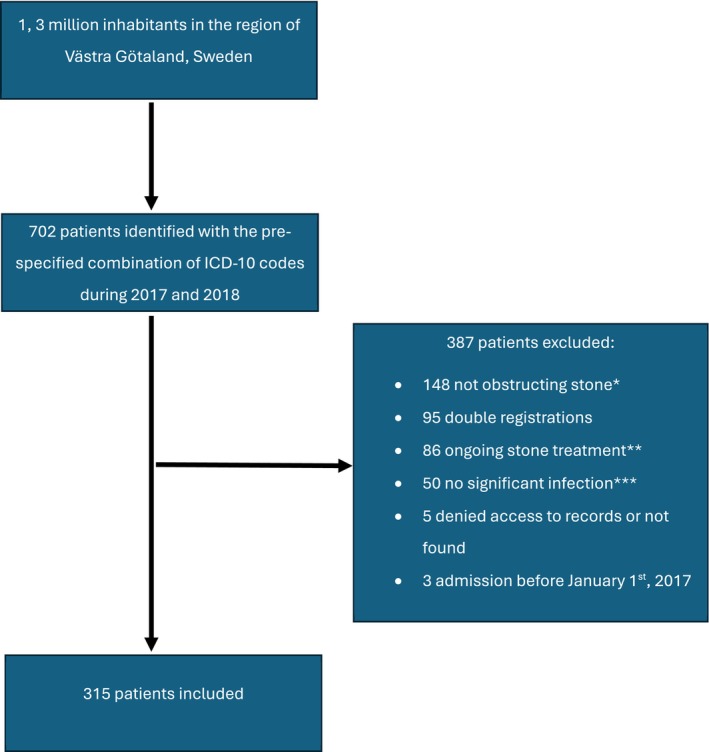
Flow diagram of patient selection. *Patients with urinary tract infection but no evidence of obstruction, for example a small peripheral calyceal stone. **Patients who underwent elective stone surgery and developed postoperative fever. ***No documentation of body temperature > 38°C or CRP‐level > 100 mg/L.

More than half of the 702 reviewed cases were excluded, most commonly due to non‐obstructing stones (Figure 1), leaving 315 patients for analysis. Based on the population in the region as of 31 December 2017, and the combined search in the registry and hospital databases, the incidence of infected obstructive upper urinary tract stones was calculated to be 11.8 per 100.000 adult inhabitants (over 18 years of age) per year (13 per 100.000 inhabitants per year for women and 10.3 per 100 000 inhabitants per year for men).

**FIGURE 2:**
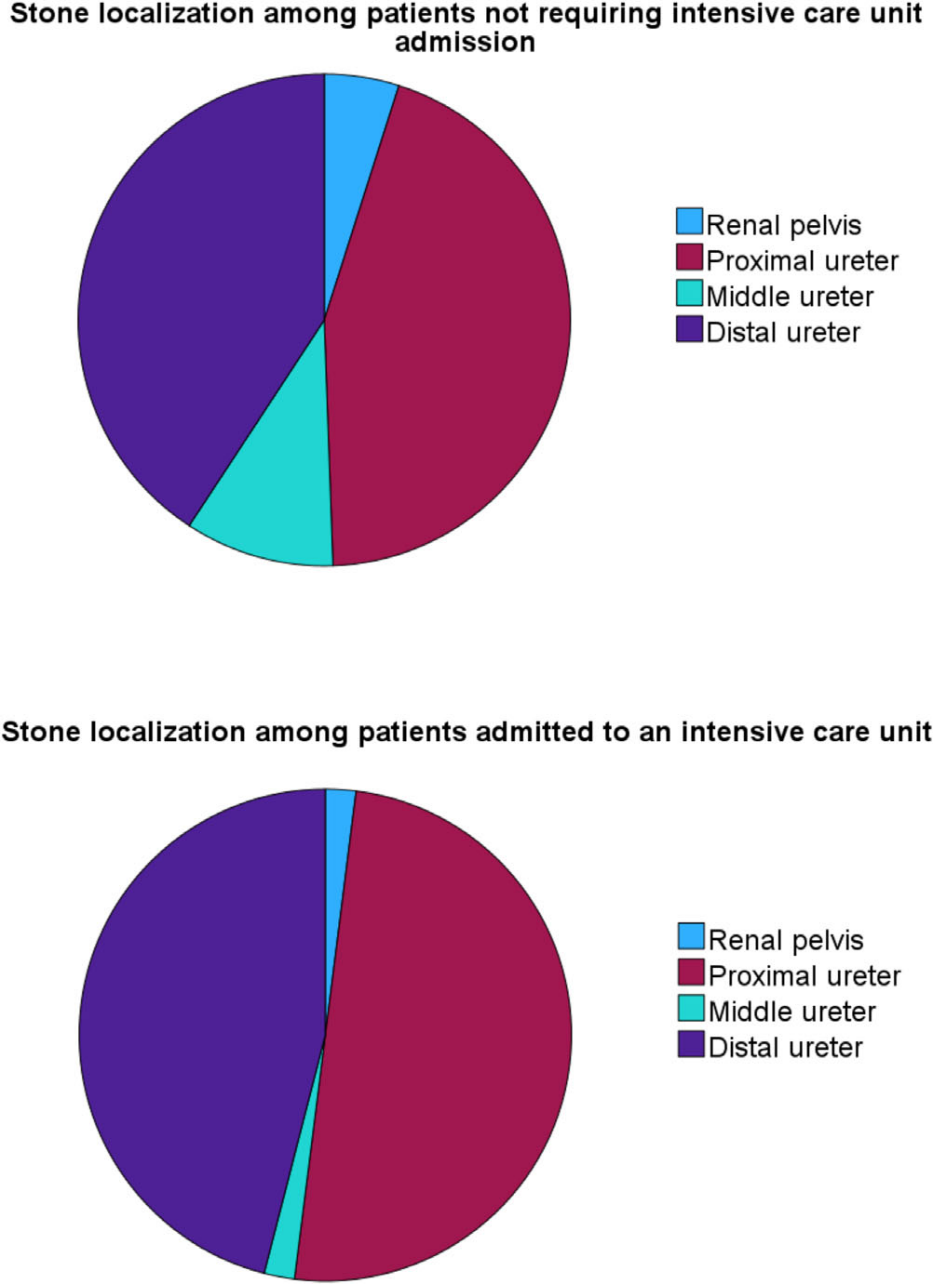
Stone localization. Comparison of patients requiring intensive care unit admission and those who did not.

The median age of the patients was 68 years (IQR 53–77), with 176 of 315 patients (56%) being female. Patient age, history of stone disease and treatment and comorbidities are shown in Table 1. Obstructing stones located in the proximal ureter were slightly more common than those in the distal ureter in all patients (Figure 2). The median stone size was 7 mm (IQR 5–9 mm) (Table 2).

**TABLE 1:** Background demographics.

Parameter	Total	Females	Males
*n*	315	176	139
Age, median (IQR)	68 (53–77)	64 (46–74)	72 (62–78)
Sex, female (%)	176 (56)	n/a	n/a
Charlson comorbidity index, median (IQR)	3 (1–5)	2 (1–4)	4 (2–6)
History of myocardial infarction (%)	18 (6)	3 (2)	15 (11)
Congestive heart failure (%)	16 (5)	5 (3)	11 (8)
Stroke (%)	26 (8)	8 (5)	18 (13)
Dementia (%)	15 (5)	7 (4)	8 (6)
Diabetes (%)	63 (20)	33 (19)	30 (22)
Kidney failure (%)	14 (4)	1 (1)	13 (9)
Risk factor UTI (%)	51 (16)	23 (13)	28 (20)
CIC^a^	8 (3)	1 (1)	7 (5)
Urethral catheter	15 (5)	4 (2)	11 (8)
Urinary diversion	2 (1)	1 (1)	1 (1)
Multiple sclerosis	6 (2)	5 (3)	1 (1)
Known ABU^b^	2 (1)	2 (1)	0 (0)
Other^c^	18 (6)	10 (6)	8 (6)
History of stone disease (%)	121 (38)	67 (38)	54 (39)
History of stone treatment (%)	67 (21)	38 (22)	29 (21)

*Note*: Percentages are within group.

^a^ Clean intermittent catheterization.

^b^ Asymptomatic bacteriuria.

^c^ History of recurrent urinary tract infections.

**TABLE 2:** All patients.

Parameter	Total	Females	Males
*n*	315	176	139
Previous visit to emergency department before admission (%)	80 (25)	45 (26)	40 (29)
SOFA score at ER presentation, median (IQR)	1 (0–2)	1 (0–2)	1 (1–2)
Hospital stay, median days (IQR)	6 (4–9)	5 (4–9)	6 (3–10)
More than one episode of hospitalization (%)	55 (17)	24 (14)	31 (22)
Deaths (%)	8 (3)	3 (2)	5 (4)
ICU^a^ admission (%)	50 (16)	33 (19)	17 (12)
Duration of symptoms^b^ until hospital admission, median days (IQR)	2 (0–4)	2 (0–4)	2 (0–5)
Duration of symptoms^b^ until decompression, median days (IQR)	3 (1–6)	3 (1–5)	3 (1–6)
Total duration with decompression until stone free, median days (IQR)	67 (30–130)	69 (35–133)	63 (27–121)
Duration admission to decompression, median days (IQR)	1 (0–1)	1 (0–1)	1 (0–2)
Patients not admitted for decompression (%)	28 (9)	19 (11)	9 (7)
Patients with decompression (%)			
PCN^c^	173 (55)	92 (52)	81 (58)
Double‐j stent	109 (34)	62 (35)	47 (34)
Ureteral catheter	5 (2)	3 (2)	2 (1)
Stone size, median millimetres (IQR)	7 (5–9)	7 (5–10)	6 (4–9)

^a^ Intensive care unit.

^b^ Flank pain or signs of infection.

^c^ Percutaneous nephrostomy.

Hospital length of stay, ED visits or readmission, details of the type of decompression administered and days until stone free are shown in Table 2. Not all patients required decompression and recovered with antibiotic therapy alone. Characteristics of this subgroup are displayed in Table 3. Notably, distal stones were more common among these patients.

**TABLE 3:** Description of patients not requiring decompression.

	Not decompressed, *n* = 28	Decompressed, *n* = 287	All patients, *n* = 315
Age, median (IQR)	63,5 (47–74)	68 (53–77)	68 (53–77)
Female sex, *n* (%)	18 (64)	158 (55)	176 (56)
CCI^a^, median (IQR)	4 (2,25–4,75)	3 (1–5)	3 (1–5)
Body temperature at the ED^b^	38 (36,9–38,6)	38 (37,2–38,9)	38 (37,2–38,9)
CRP at the ED, median (IQR)	72 (19–176)	110 (41–209)	110 (40–204)
Stone localization, *n* (%)			
Renal pelvis	4 (14)	10 (3)	14 (4)
Proximal ureter	2 (7)	141 (49)	143 (45)
Middle ureter	2 (7)	25 (9)	27 (9)
Distal ureter	20 (71)	111 (39)	131 (42)
Stone diameter, mm (IQR)	4 (3–6)	7 (5–10)	7 (5–9)
ICU admission, *n* (%)	1 (4)	49 (17)	50 (16)

^a^ Charlson comorbidity index. IQR = interquartile range.

^b^ Emergency department.

**FIGURE 3:**
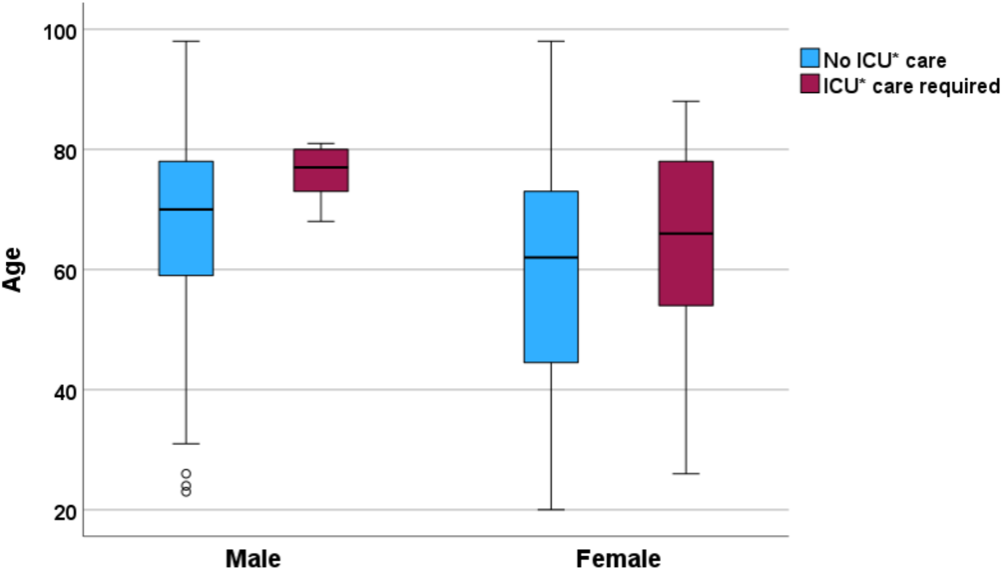
Comparison of age distribution among patients requiring intensive care unit admission and those who did not. *ICU = intensive care unit.

Intensive care unit (ICU) admission was required in 50 cases (16%), and there were 8 deaths (3%). The median length of stay in the ICU was 1 day (IQR 1–3), with one patient who had severe comorbidities requiring 61 days. ICU patients were predominantly female (33 cases, 66%), they were generally younger than their male counterparts (median age 77 years for men and 66 years for women; Figure 3) and had a lower CCI (median 3 for women compared to 6 for men).

Details of the eight mortalities are listed in a Supporting Information table. All deceased patients were ≥ 70 years of age. Five deaths were directly attributable to septic complications, while the remaining three were due to the deterioration of pre‐existing conditions while awaiting definitive stone treatment.

Three patients did not receive definitive treatment for the obstructing stone and were managed with lifelong decompression. One of these patients later died, although the cause of death was not documented. The other two patients had severe comorbid conditions, one with advanced sequelae after a cerebrovascular insult (CVI) and the other with a severe psychiatric disorder that rendered surgical or shockwave treatment infeasible.

## 4 DISCUSSION

In this study, we found an annual incidence of 11.8 per 100.000 inhabitants per year that were hospitalized because of a combination of obstructing upper urinary tract stones and UTI. This figure shows that this is not an uncommon condition and that 16% needed intensive care facilities demonstrate its clinical seriousness. The median age was quite high (68 years), and as the general population is expected to include an increasing number of elderly people over time, this clinical condition will probably also become more common. For 2018, the incidence in our region of kidney stones diagnosed (all N20.0 to N20.9) was 275 per 100.000 inhabitants^13^ indicating that around 4% of patients with upper tract stones are affected.

The annual incidence of infected obstructive stones described here is slightly lower than that previously reported by Sammon et al. in the United States (20 per 100.000 inhabitants in 2009).^10^ However, we have employed a different methodology with examination of individual patient records, and this showed to exclude a significant number of cases with no proven infection possibly indicating that ICD‐10 codes alone might not capture the correct number of patients.

This study confirms that although most patients were given adequate initial treatment, the combination of obstructing urinary stones and UTI is still a lethal condition despite access to ICUs, modern antibiotics, and 24‐hour availability for upper urinary tract drainage. All the deceased patients were aged >70 years. Five had previous serious comorbidities that contributed to their deaths, but three of eight were healthy individuals who died from acute septic complications. Sepsis and septic shock are known to cause significant mortality,^14^ but studies on this particular subgroup of urosepsis cases are lacking, making comparisons with our study population difficult.

On the contrary, 9% (28 patients) did not require decompression at all and were treated with antibiotic therapy alone, illustrating the heterogeneity of this clinical condition. These patients were often planned for intervention, but it was delayed due to practical reasons (operation theatre or interventional radiologist not available) and upon renewed examination showed clinical improvement. They had mostly distal ureteric stones of smaller size, which are more likely to pass spontaneously, and this is probably the reason for their recovery. This emphasizes that stone removal/passage is also a good drainage, and these findings may indicate that some patients can be treated conservatively. Close surveillance is of course necessary as the clinical status can rapidly deteriorate, and previous studies have shown an increased risk of death when decompression is delayed.^9^, ^15^

A liberal indication for early decompression is recommended in the European Association of Urology Guidelines, although the panel concludes that the evidence is mostly of lower quality.^16^ According to the guidelines of the American Association of Urology, the recommended treatment for upper tract stones and UTI with or without sepsis is drainage and antibiotic therapy; however, there are no further details on the possibility of differentiating treatment depending on individual patient properties.^17^, ^18^, ^19^ Further studies focusing on this subgroup of patients are of great importance before any recommendations can be made to deviate from current practice with rapid decompression of all patients with suspected obstructive infection.

The median waiting time for stone treatment and removal of upper tract drainage was 67 days, which reflects the long waiting time in the health care system. This long waiting time for definite stone treatment was associated with significant morbidity; 17% of patients required more than one episode of hospital admission. Further studies on optimal treatment and timing of treatment are needed, as this appears to be a subgroup of stone patients who are at risk of prolonged suffering and increased health care costs.

We found that women and men were equally often admitted because of infections associated with obstructing stones. Considering that men have at least a two‐fold higher risk of upper tract stone disease,^20^ this confirms that women are more susceptible to infectious complications. Especially among patients requiring ICU admission, females were younger and had a lower comorbidity index at ER presentation than males. This is in line with previous findings from the United States and Great Britain.^2^, ^10^, ^21^, ^22^ The underlying cause of this is unrecognized, but it has been proposed that it simply reflects the higher prevalence of UTIs in women than in men.^23^ Infection‐associated stones are also more common in women, probably because of their increased risk of UTIs.^24^ Furthermore, women are at a higher risk of death from severe sepsis/septic shock.^25^ The reason for this is still unknown and further stresses the need for research in this field.

There are several strengths and weaknesses of this study. The Swedish registers have, in general, a high validity^26^ and give us a unique possibility to calculate a population‐based estimation of the burden of this clinical condition. However, in this study, we found that the Swedish National Patient register was unreliable, and many patients were incorrectly registered or reported. By also searching local hospital databases, we hoped to minimize the risk of missing patients who were treated in our region. As the population register in Sweden allows us to find patients even though they have moved or changed their surnames, we believe that the number of patients not identified is low. We used a wide search criterion in an attempt to minimize missed cases, but the incidence number presented in this study must be considered with the search limitation in mind and seen as an approximation.

Also, we have not used the Sepsis‐3 definition for sepsis in the inclusion criteria as this was considered a risk to miss cases presenting without sepsis but who will soon develop severe infection. Furthermore, exclusion of our approximation for insignificant infections (no record of fever ≥38° C or CRP > 100) might also carry a risk, as elderly or immuno‐compromised patients may react differently even with severe infection. Our approach was pragmatic as we hope the results will aid clinical decisions but is a limitation as a strict use of sepsis criteria might make results more applicable at other centers. Also, no urine or blood culture results were available in this study which could aid in identifying infections originating from the urinary tract.

Another limitation of the current study is its retrospective design. There is a risk of bias when data are collected for reasons other than research, and retrospective data are always subject to interpretation by the researchers. Future prospective studies are warranted, and the current results can serve as a basis for such.

There is a further challenge in identifying this group as there is a lack of a specific ICD‐10 code for this entity. Most urologists worldwide recognize these patients in the clinical setting, but there is no clear diagnostic classification. In many studies, the term ‘pyonephrosis’ (N13.6 in the ICD‐10 classification) is used. However, this describes a suppurative infection in the renal pelvis, creating pus, which is not always present, and conversely, may be present without concomitant systemic infection/sepsis. The terms ‘infected obstructive hydronephrosis’ or ‘acute obstructive pyelonephrosis’ may be more correct but lack a separate ICD‐10 code. In a publication from 2020 in Nature Reviews Nephrology, the authors discussed the need for better classification of all kidney stones.^27^ It would be of great importance for future research that we also classify the patients analysed in the current study correctly and in more detail.

In this study, we settled for the above selection of codes as this is the routine administrative practice used by all medical secretaries in our region. However, individual deviations may of course exist and would result in missed cases.

## 5 CONCLUSIONS

This retrospective analysis of patients with infected upper tract stones shows that it is a variable disease. It affects both men and women, with significant morbidity and even mortality. There is a significant challenge in identifying these patients through studies using the existing ICD‐10 codes.

## AUTHOR CONTRIBUTIONS

*Conception and design:* H.S., J.H., L.G., K.L., E.S. *C* *ritical revision of the manuscript for scientific and factual content:* J.H., L.G., K.L., E.S. *Drafting the manuscript:* H.S., J.H. *Data analysis and interpretation:* H.S., J.H., L.G., K.L., E.S. *Data acquisition:* H.S. *Statistical analysis:* H.S. *Supervision:* J.H., L.G., K.L., E.S.

## CONFLICT OF INTEREST STATEMENT

The authors have no conflicts of interest to disclose.

## Supporting information


**Table S1:** Description of the eight patients who died during the study period (from admission to hospital until stone free).
