# Supplementary material for: Urosepsis due to obstructive stones: Epidemiological data from a population‐based study in Sweden
Source: BJUI Compass. 2026 Feb 16;7(2):e70158. doi: 10.1002/bco2.70158 (PMC12908422; doi:10.1002/bco2.70158)
Supplement: Supplementary file 1 — Table S1: Description of the eight patients who died during the study period (from admission to hospital until stone free). [file BCO2-7-e70158-s001.docx]

Supplementary table: Description of the eight patients who died during the study period (from admission to hospital until stone free).

| *Patient nr* | *Age* | *CCI* | *Sex* | *Days from admission to death* | *Days from 1^st^ symptom to decompression* | *Cause of death* |
| --- | --- | --- | --- | --- | --- | --- |
| 1 | 79 | 7 | male | 41 | 2 | Retroperitoneal hemorrhage when changing dysfunctional nephrostomy tube. |
| 2 | 83 | 8 | male | 9 | 1 | Circulatory and respiratory failure caused by sepsis. |
| 3 | 79 | 4 | female | 11 | 1 | Multiorgan failure caused by sepsis. |
| 4 | 82 | 9 | male | 168 | 1 | Gastrointestinal hemorrhage while waiting for percutaneous stone surgery. |
| 5 | 83 | 6 | female | 153 | 2 | Neurologic deterioration due to other reasons while waiting for ureteroscopy. |
| 6 | 86 | 4 | female | 188 | 4 | Heart failure, myocardial infarction while waiting for ureteroscopy. |
| 7 | 76 | 7 | male | 39 | 1 | Previous multiple sclerosis. Respiratory failure caused by sepsis. |
| 8 | 72 | 5 | male | 29 | 2 | Previous multiple sclerosis and ischemic heart disease. Heart failure caused by sepsis. |
